# Missing link between tissue specific expressing pattern of ERβ and the clinical manifestations in LGBLEL

**DOI:** 10.3389/fmed.2023.1168977

**Published:** 2023-06-29

**Authors:** Xujuan Zhang, Pengxiang Zhao, Mingshen Ma, Hao Wu, Rui Liu, Ziyi Liu, Zisong Cai, Mengyu Liu, Fei Xie, Xuemei Ma

**Affiliations:** ^1^Faculty of Environment and Life, Beijing University of Technology, Beijing, China; ^2^Beijing Molecular Hydrogen Research Center, Beijing, China; ^3^Beijing International Science and Technology Cooperation Base of Antivirus Drug, Beijing, China; ^4^Department of Ophthalmology, Beijing Chaoyang Hospital, Capital Medical University, Beijing, China; ^5^Beijing Tongren Hospital, Capital Medical University, Beijing, China

**Keywords:** LGBLEL, ERβ, B lymphocyte proliferation, lacrimal gland apoptosis, clinical manifestations, tumor development

## Abstract

**Purpose:**

Lacrimal gland benign lymphoepithelial lesion (LGBLEL) is an IgG4-related disease of unknown etiology with a risk for malignant transformation. Estrogen is considered to be related to LGBLEL onset.

**Methods:**

Seventy-eight LGBLEL and 13 control clinical samples were collected and studied to determine the relationship between estrogen and its receptors and LGBLEL development.

**Results:**

The serological analysis revealed no significant differences in the levels of three estrogens be-tween the LGBLEL and control groups. However, immunohistochemical analyses indicated that the expression levels of ERβ and its downstream receptor RERG were relatively lower in LGBLEL samples than in control samples, with higher expression in the lacrimal gland and lower expression in the lymphocyte infiltration region. However, low expression of ERα was detected. The transcriptome sequence analysis revealed upregulated genes associated with LGBLEL enriched in lymphocyte proliferation and activation function; downregulated genes were enriched in epithelial and vascular proliferation functions. The key genes and gene networks were further analyzed. Interactions between B cells and epithelial cells were analyzed due to the identified involvement of leukocyte subsets and epithelial cells. B cell proliferation was found to potentially contribute to lacrimal gland apoptosis.

**Conclusion:**

Therefore, the tissue-heterogeneous expression pattern of ERβ is potentially related to the clinical manifestations and progression of LGBLEL, although further investigations are required to confirm this finding.

## 1. Introduction

Lacrimal gland benign lymphoepithelial lesion (LGBLEL), also known as Mickulicz’s disease, is characterized by the diffuse infiltration of lymphocytes and plasma cells into the lacrimal gland tissue, the atrophy and disappearance of glands, and excessive fibrosis (1, 2). Typical symptoms of LGBLEL are bilateral swelling of eyelids and diffuse enlargement of the lacrimal glands, potentially related to immunoglobulin G4 (IgG4)-positive plasma cell infiltration into the affected tissues (3, 4). The etiology of this disease remains unclear; however, B cell proliferation and BCR signaling activation (1) could contribute to lacrimal gland tissue hypoplasia and IgG4 production, similar to the effects observed in Sjogren’s syndrome (5).

Clinically, LGBLEL tends to affect middle-aged women, whose estrogen levels are declining, especially after menopause (6). Therefore, it is hypothesized that LGBLEL pathogenesis arises from a sex hormone imbalance (7). The three major forms of estrogens are estrone (E1), estradiol (E2), and estriol (E3), among which estradiol (E3) is predominant in non-pregnant women (8). Estrogens mainly act by binding to two specific receptors (ERs), ERα and ERβ (8–10). ERα has been comprehensively studied for several decades, and it is predominantly expressed in major organs, such as most female reproductive organs (9), the liver (11), the pituitary gland (12), and the hypothalamus (12). ERα is also expressed in bones (13). However, ERβ is expressed in fewer tissues, such as the ovary (14), lung (11), adult cerebellum (15), and gastrointestinal tract (11).

ERα has been well-studied in tumorigenesis, and ERβ has been found to be involved in tumor development regulation. Specifically, ERα typically acts as an oncogene, while ERβ is a tumor suppressor (16). ERβ has been shown to be expressed at significantly higher levels in normal ovarian tissues than in ovarian carcinomas (17, 18). ERβ is the predominant ER expressed in colon tissues, and its loss has been associated with advanced colon cancer (19). Notably, the ER receptor pathway is also involved in B lymphocyte function enhancement, representing a promising target for anti-autoimmunity or anti-tumor therapy (20). Though normal human peripheral blood cells express both ERα and ERβ, B-cell malignancies express mainly ERβ (21), and selective ERβ agonists inhibit cell growth and induce apoptosis (21). Therefore, ERβ could play an important role in B cell tumorigenesis. Estrogens deficiency could lead to the lacrimal gland apoptosis, necrosis, and lymphocytic infiltration (22). As ERβ has been shown to be predominantly expressed in the lacrimal gland (23), and involved in the functions of lymphocytes, especially B cells, it might also be involved in LGBLEL pathogenesis.

In our study, by serological, histological, and transcriptome analyses of LGBLEL clinical samples, we uncovered the possible link between the multiple functions of ERβ and LGBLEL. We found no significant changes in whole-body estrogen levels or ERα expression levels in affected tissues in LGBLEL patients. However, the ERβ expression was observed to be tissue specific. In the lacrimal gland region of LGBLEL, ERβ was highly expressed, while relatively lower expressed in the lymphocytic infiltration region. These findings may explain the clinical features of lymphocyte proliferation and infiltration and lacrimal gland atrophy in LGBLEL, providing clues for the clinical diagnosis and treatment of LGBLEL.

## 2. Materials and methods

### 2.1. Study subject

Orbital tissue biopsy and blood serum from patients with LGBLEL, CH, and LGP were collected immediately after surgery. A portion of the collected tissues was stored in liquid nitrogen until subsequent assays; the rest of the collected tissues were fixed in formalin, embedded in paraffin, cut into 5 μm sections for downstream histological analysis. The collected serum was stored in −80°C for further assessment.

### 2.2. Immunohistochemistry (IHC)

For IHC staining, sections were deparaffinized and rehydrated with a histoclear/alcohol series and antigen retrieval was performed by boiling in 1 × citrate antigen retrieval buffer (Vector), endogenous peroxidases were blocked by treating slides with 3% hydrogen peroxide for 15 min at room temperature. Slides were blocked in PBS with 5% normal goat serum (Bioss, C0005) and primary antibody staining was performed overnight in PBS with 1% goat serum at 4 °C. The following primary antibodies were used: Mouse anti-ER alpha (ESR1, 1:200, Novus, NB200-560), Mouse anti-ER beta (ESR2, 1:200, Novus, NB200-305), Mouse anti-CD19 (1:1,000, Proteintech, 66298-1-Ig), Mouse anti-Ki67 (1:1,000, CST, 9449), Rabbit anti-RERG (1:20, Proteintech, 10687-1-AP), and Anti-8-OHdG (1:200, Bioss, 1278R). Further labeling with specific secondary antibodies for DAB staining was performed using the HRP-conjugated Goat Anti-Rabbit/Mouse IgG Polymer Peroxidase Detection Kit (ZSGB-BIO, PV-6001/6002) for 1 h at room temperature and developed with DAB reagent (CST, 80059S). Sections were counterstained with haematoxylin (ZSGB-BIO, ZLI-9610) and mounted with neutral gum (ZSGB-BIO, ZLI-9555) after dehydration.

Semiquantitative IHC analysis of ERα ERβ and RERG expression was conducted by the H-scoring system. The H-score was calculated as: 3 × percentage contribution of High Positive + 2 × percentage contribution of Positive + 1 × percentage contribution of Low positive. The percentage contribution was analyzed using IHC Profiler of ImageJ software (ver. 2.1.0). The total H-score per sample therefore ranged from 0 to 300. H-scores were classified as negative (0–50), weakly positive (51–100), moderately positive (101–200), or strongly positive (201–300). At least 5 random visual fields were counted for each group and then differences were analyzed using *t*-test.

### 2.3. Immunofluorescent (IF)

For immunofluorescence analysis, after deparaffinization, rehydration and antigen retrieval. Slides were blocked in PBS with 5% normal goat serum (Bioss, C0005) and were incubated overnight at 4°C with the following antibodies: Rabbit anti-pan-keratin (1:400; Proteintech; 26411-1-AP), Mouse anti-ER beta (1:200, Novus, NB200-305), Rabbit anti-Caspase3 (1:50; Proteintech; 19677-1-AP), and Mouse anti-CD27 (1:1,000, Proteintech, 66298-1-Ig). The sections were next incubated with the following fluorescent secondary antibodies for 1 h at room temperature (Alexa Fluor^®^ 488 Conjugate anti-rabbit IgG (H + L), 1:1,000, CST, 4412S; Alexa Fluor^®^ 594 Conjugate anti-mouse IgG (H + L), 1:500, CST, 8890S). Finally, the nuclei were stained with 4′,6-diamidino-2-phenylindole (DAPI) and the sections were examined with a fluorescence microscope. The percentage of positive cells staining positively was analyzed using ImageJ software (ver. 2.1.0).

### 2.4. Enzyme-linked immunosorbent assay

The following commercial immunoassay kits were used to detect hormone levels present in plasma samples: (estriol, Novus, NBP2-61289; estrone, Abnova, KA1908; estradiol, Abnova, KA0234). A curve-fitting statistical software was used to plot a 4-parameter logistic curve fit to the standards and then calculate results for the all the samples according to the manufacturer’s instructions.

### 2.5. RNA extraction and quantitative RT-PCR

Total RNA from the collected tissues was isolated with TRIzol reagent (Invitrogen, 15596018). Reverse transcription was performed using the ReverTra Ace^®^ qPCR RT kit (TOYOBO, FSQ-101). Quantitative RT-PCR (qRT-PCR) was performed using the SYBR^®^ Green Realtime PCR Master Mix (TOYOBO, QPK-201). Primer used for qRT-PCR were: RERG forward, 5-TGGTCTACGACATTACTGACCG-3 and reverse, 5-AAGCACAAGCCAATTCTGTGG-3; MMP2 forward, 5-GATACCCCTTTGACGGTAAGGA-3 and reverse, 5-CCTTCT CCCAAGGTCCATAGC-3; VEGFA forward, 5-AGGGCAGAAT CATCACGAAGT-3, and reverse, 5-AGGGTCTCGATTGGAT GGCA-3; PCNA forward, 5-ACACTAAGGGCCGAAGATAACG-3 and reverse, 5-ACAGCATCTCCAATATGGCTGA-3; MMP9 forward, 5-TGTACCGCTATGGTTACACTCG-3 and reverse, 5-GGCAGGGACAGTTGCTTCT-3; and reference gene GAPDH forward, 5-GAAATCCCATCACCATCTTCCAGG-3 and reverse, 5-GAGCCCCAGCCTTCTCCATG-3.

### 2.6. Microarray analysis

Orbital CH and BLEL tissue biopsies microarray data deposited in gene expression omnibus by Wang and Ma (7) under the accession number GSE76497 were used for genes expression profiling and for functional annotation. Background correction, quantile normalization and summarization of the expression data were performed under GeneSpring version 14.9 (Agilent Technologies). The significant DEGs were selected with a *p*-value < 0.05 and | FC| ≥ 1. The heatmap was plotted using the “pheatmap” package through the software “R” and the integrated development environment “RStudio” (24), packages found in the repositories CRAN (“The Comprehensive R Archive Network”) (25) and Bioconductor (26) and Z-score normalization (27) was calculated in the heatmap. For functional enrichment analysis, the Metascape (28) web tool was used to query GO processes and KEGG pathway. Protein-Protein Interaction analysis was performed in STRING database (29), CIBERSORT (30) was used to analysis leukocyte subsets through the “R” and the “RStudio,” different cell states in LGBLEL epithelial cells and interactions between B cells and epithelial cells were specifically identified by the EcoTyper web tool (31) based on tissue expression profile, gene functional enrichment was performed by Gene Set Enrichment Analysis (GSEA) (32).

### 2.7. Statistical analysis

Quantitative data were analyzed using GraphPad Prism v.9 (GraphPad Software, Inc., La Jolla, CA, USA) and are plotted as the mean ± standard error of the mean. For group comparisons, one-way analysis of variance with Bonferroni’s multiple comparison test was used. *P* < 0.05 was considered to indicate a statistically significant difference.

## 3. Results

### 3.1. Clinical features and histological features

A total of 78 LGBLEL and 13 orbital cavernous hemangioma (CH) patients recruited between March 2015 and November 2020 were included in this study. The LGBLEL patient sex ratio was 3.59:1 women to men, and the median age was 48 years (range, 12–70 years; SE, 1.31); the CH patient sex ratio was 6.50:1 women to men, and the median age was 52 years (range, 34–77 years; SE, 3.95). Swelling of lacrimal glands was observed in all study patients, with eyelid swelling in both eyes in approximately 69.23% (54/78) of LGBLEL patients and 7.69% (1/13) of CH patients, which continued for over 6 months in most patients (50/78, 64.10% in LGBLEL; 10/13, 76.92% in CH) (Table 1).

**TABLE 1:** Summarized clinical information of the study population.

Study group	LGBLEL		CH	
	**Mean**	**SE**	**Mean**	**SE**
Males/Females	17/61		2/11	
Age (Years)	48 (12–70)	1.31	52 (34–77)	3.95
**Eyelid swelling**				
Both	54		1	
Left	12		6	
Right	12		6	
**Swelling duration**				
≤ 6 months	28		3	
7–12 months	23		2	
> 12 months	27		8	

### 3.2. Histopathological features and expression levels of estrogen receptors in LGBLEL samples

**FIGURE 1:**
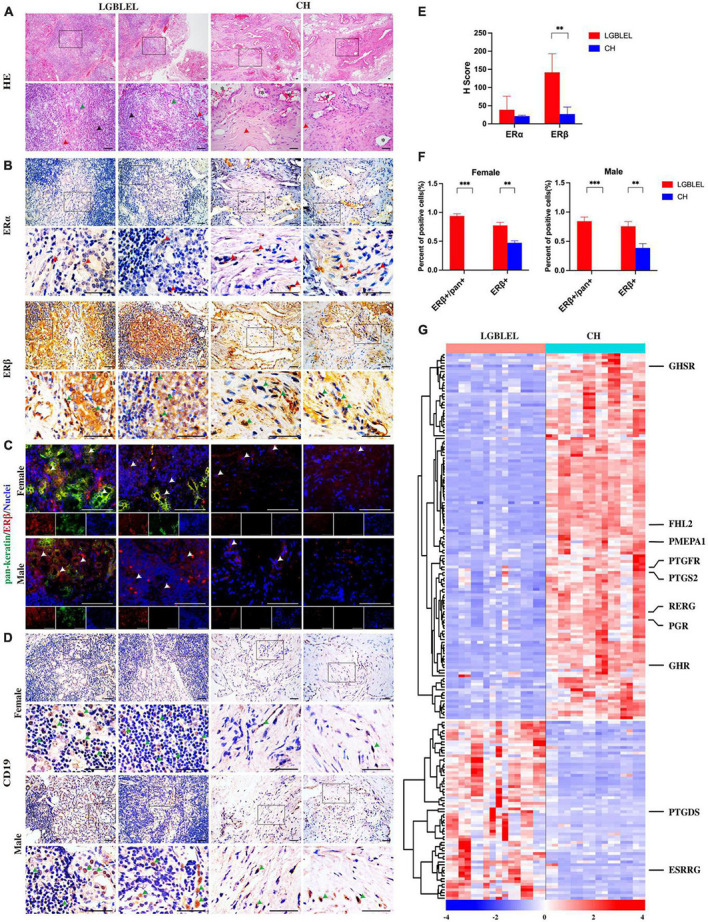
**(A)** Hematoxylin and eosin staining showing lymphocytic infiltration in LGBLEL tissues (black arrows), acinar atrophy (green arrows), and the fibrotic area (red arrows). In the CH panel, *indicates dilated blood vessels, while the red arrows indicate cells with fibroblast-like morphology; **(B)** Immunohistochemistry results for estrogen receptors ERα (red arrows) and ERβ (green arrows) in LGBLEL and control CH; **(C)** Fluorescence co-localization results of ERβ (red) and the lacrimal gland marker pan-keratin (green); **(D)** The expression of B lymphocyte marker CD19 in lymphatic cell-infiltrated areas of LGBLEL; **(E)** The quantitative determination of ERα and ERβ IHC staining; **(F)** The quantitative determination of the fluorescence co-localization results of ERβ and pan-keratin; **(G)** Heatmap of gene expression levels in the microarray data related to responses to hormones (GO: 0009725). Scale bar: 20 μm. ***p < 0.001, **p < 0.01.

Histological analysis of paraffin-embedded tissue sections using hematoxylin and eosin (H&E) staining (Figure 1A) showed acinar atrophy, massive lymphocyte infiltration, and different degrees of fibrosis in all LGBLEL specimens. In the control group comprising patients with orbital cavernous hemangioma (CH), a type of blood vessel malformation in which a collection of dilated blood vessels forms a benign tumor, a higher degree of fibrosis and absence of infiltrates were notable.

To characterize the plasma levels of estrogen in the female LGBLEL and CH patients, we first assessed the plasma levels of estrone, estradiol, and estriol by enzyme-linked immunosorbent assay (ELISA) and then divided the patients by age (20–40 years and over 40 years). In addition, the relative expression levels of genes involved in estrogen biosynthesis were analyzed by microarray (Supplementary Figure 1). Our results showed no significant differences in estrone, estriol, or estradiol levels in LGBLEL samples compared to those in CH samples (Supplementary Figure 1A), and no significant age-related differences were observed (Supplementary Figure 1B).

To further explore the response to estrogen in LGBLEL, we first detected the expression of estrogen receptors. The IHC results showed that ERβ [encoded by the estrogen receptor 2 (ESR2) gene] was observed in almost all lacrimal glands and some lymphatic cell-infiltrated areas, ERα [encoded by the estrogen receptor 1 (ESR1) gene] was rarely expressed in the lacrimal glands or lymphatic cell-infiltrated areas in LGBLEL, while ERα and ERβ were both expressed in the vessel wall and fibrotic areas in CH (Figure 1B). According to the H-Score system, the expression of ERβ in LGBLEL was significantly higher than that in CH, and the expression of ERβ in LGBLEL was moderately positive (100 < H-score < 200), which is negative in CH (H-score < 50); ERα expression in both groups was weak positive/negative (H-score < 100) (Figure 1E). To further confirm the expression of ERβ in the lacrimal gland region and whether it is affected by gender, we examined the fluorescence colocalization of the lacrimal gland marker pan-keratin with ERβ in female and male LGBLEL and CH. In LGBLEL, ERβ was expressed at the location of pan-keratin fluorescence signal in female and male, while in CH, there was no pan-keratin fluorescence signal due to the absence of the lacrimal gland. Fluorescence signals for ERβ were also detected in lymphatic cell-infiltrated areas (Figure 1C). The ratio of ERβ^+^/pan^+^ in pan^+^ cells and ERβ^+^ cells in all the observed areas were calculated. The results showed that the ratio of ERβ^+^/pan^+^ was high in both female and male LGBLEL. Moreover, ERβ^+^ was significantly higher in both male and female LGBLEL than in the CH (Figure 1F).

The expression of B lymphocyte marker CD19 confirmed the presence of B lymphocyte infiltration of LGBLEL (Figure 1D).

In addition to evaluating the expression of the two estrogen receptors, ERα and ERβ, we simultaneously examined gene expression levels of response to hormone (GO: 0009725) using microarray data. The heatmap revealed that the expression of 130 genes was downregulated and that of 64 genes were upregulated in LGBLEL compared to that in CH (Figure 1C), among which, growth hormone secretagogue receptor (GHSR), four and a half LIM domains 2 (FHL2), prostate transmembrane protein, androgen induced 1 (PMEPA1), prostaglandin F receptor (PTGFR), prostaglandin-endoperoxide synthase 2 (PTGS2), RAS-like estrogen regulated growth inhibitor (RERG), progesterone receptor (PGR), and growth hormone receptor (GHR) were downregulated in LGBLEL, while other genes, such as prostaglandin D2 synthase (PTGDS) and estrogen-related receptor beta (ESRRB) were upregulated.

For further validation, we examined the expression of ERα and ERβ in the lacrimal gland tissues of patients with lacrimal gland prolapse, an estrogen-independent lesion of the lacrimal gland without lymphatic infiltration, and found that ERα expression was absent and ERβ was expressed at low levels (Supplementary Figure 2).

Based on these results, we investigated the expression of many hormone receptors and proliferation-regulating genes were downregulated in LGBLEL. ERβ was mainly expressed in the lacrimal glands and relatively lower expressed in the areas of lymphocyte infiltration. Thus, high ERβ expression may be a characteristic of LGBLEL and may have different effects on the lacrimal gland and lymphocytes; the inhibition of cell proliferation may be critical in this process.

### 3.3. Analysis of cell proliferation characteristics in LGBLEL

**FIGURE 2:**
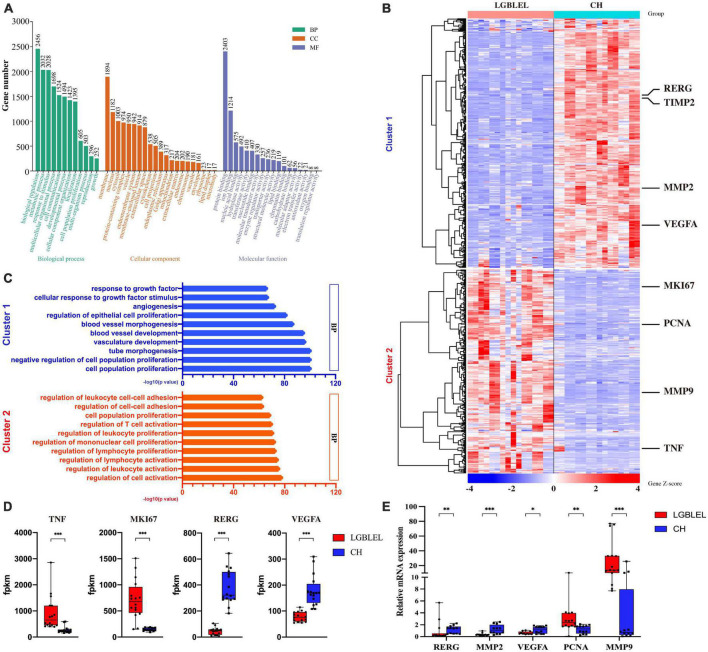
**(A)** Enriched GO slim terms among differentially expressed genes based on expression profile microarray; **(B)** Heatmap of the 625 genes enriched in cell population proliferation of biological process in the GO slim; **(C)** The 10 most significant terms of enriched gene ontology in clusters 1 and 2 of the heatmap; **(D)** Histogram of the FPKM values of the representative genes in the heatmap comparison of LGBLEL and CH; **(E)** Expression levels of tissues mRNA of the representative genes in the heatmap comparison of LGBLEL and CH. *P*-values were calculated using a Mann–Whitney test. ***p < 0.001, **p < 0.01, and *p < 0.05.

To better understand the characteristics of lymphocyte infiltration and lacrimal gland atrophy in LGBLEL, we analyzed the DEGs based on expression profile microarray for Gene Ontology (GO) and KEGG terms using Metascape the top 10 significant terms are shown in Supplementary Table 1 and Supplementary Figure 3 and performed a GO slim enrichment analysis using WebGestalt to identify significant biological functions (Figure 2A). Then, 625 genes enriched in cell population proliferation in the biological process (BP) subset were further analyzed by a heatmap, revealing two clusters; in cluster 1, 337 downregulated genes were present in LGBLEL samples compared to CH samples, and in cluster 2, 268 upregulated genes were present (Figure 2B). A GO BP enrichment analysis was performed on the genes in clusters 1 and 2, and the 10 most significant terms are shown in Figure 2C; most of the terms related to epithelial and vascular proliferation were enriched in cluster 1, while most of the terms related to lymphocyte proliferation or activation were enriched in cluster 2. Meanwhile, we also analyzed the fragments per kilobase of transcript per million mapped reads (FPKM) values of several representative genes. The expression levels of TNF and MKI67 in LGBLEL were significantly higher than those in CH, and the expression levels of RERG and VEGFA in LGBLEL were significantly lower than those in CH (Figure 2D). To further confirm these results, we examined the mRNA expression levels of several representative genes in the tissues. The expression levels of RERG, MMP2 and VEGFA in LGBLEL were significantly lower than those in CH, and PCNA and MMP9 in LGBLEL were significantly higher than those in CH (Figure 2E).

**FIGURE 3:**
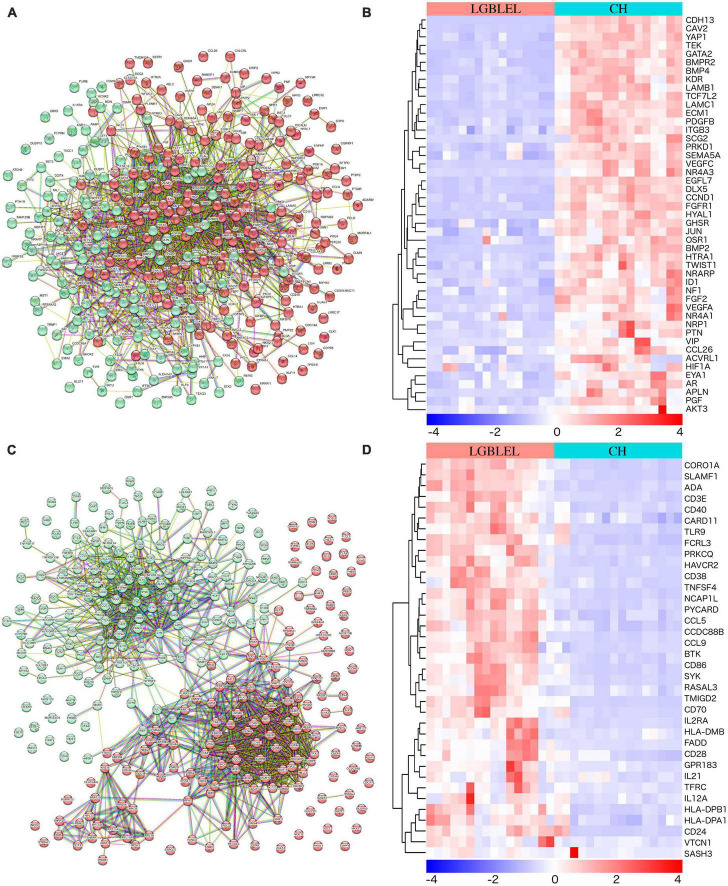
**(A)** Protein–protein interaction analysis of 337 downregulated genes; **(B)** Heatmap of the 47 genes were enriched in positive regulation of epithelial cell proliferation (GO: 0050679); **(C)** PPI analysis of 268 upregulated genes; **(D)** Heatmap of the 37 genes were enriched in positive regulation of lymphocyte proliferation (GO: 0050671).

We observed downregulated genes with functions such as epithelial cell proliferation in LGBLEL compared to CH and upregulated genes associated with lymphocyte proliferation; therefore, to further study the proliferation characteristics of epithelial cells and lymphocytes in LGBLEL, we performed a protein–protein interaction (PPI) analysis of 337 downregulated genes in cluster 1 (Figure 3A), which revealed that 47 genes were enriched in GO: 0050679 (positive regulation of epithelial cell proliferation) (Figure 3B). The PPI analysis of 268 upregulated genes in cluster 1 (Figure 3C) revealed that 37 genes were enriched in GO: 0050671 (positive regulation of lymphocyte proliferation) (Figure 3D). As shown in Figure 3C, the 268 upregulated genes were clearly divided into two clusters: B cell-related (cyan-colored ball) and proliferation-related (red-colored ball) genes. GSEA enrichment analysis further confirmed that up-regulated genes in LGBLEL were related to immune processes, while down-regulated genes were related to angiogenesis processes the top 5 significant GSEA GO and KEGG terms are shown in Supplementary Figure 4 and Supplementary Table 2.

### 3.4. The characteristics of immune cell and epithelial cell characteristics in LGBLEL

**FIGURE 4:**
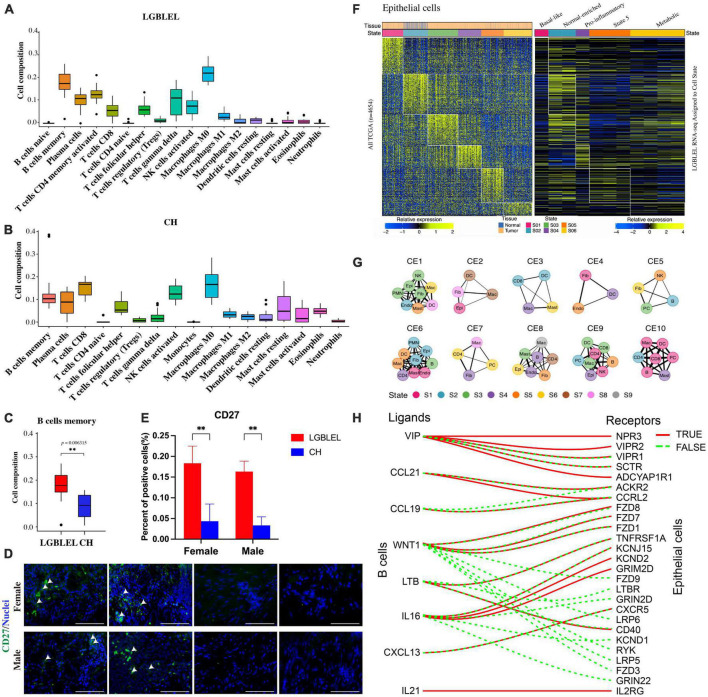
Analysis of immune cell subsets and epithelial cell states using expression profile microarray data: **(A)** Immune cell subsets of LGBLEL; **(B)** Immune cell subsets of CH; **(C)** Cell composition of B cells memory in LGBLEL vs. CH; **(D)** Tissue immunofluorescence results of memory B cell surface marker CD27 in LGBLEL and CH; **(E)** The quantitative determination of the tissue immunofluorescence results of CD27; **(F)** The left heatmap is displayed as a reference to depict the epithelial-specific expression of cell state signature genes across The Cancer Genome Atlas (TCGA) carcinoma samples. The right heatmap depicting the epithelial specific expression of cell state signature genes in the LGBLEL data; **(G)** Cell state networks for each ecotype (31); **(H)** B cell ligand and epithelial cell receptor enrichment analysis. **P < 0.006315.

To further analyze the characteristics of immune cells in LGBLEL, we used CIBERSORT, a computational framework that accurately infers cell-type abundance and cell type–specific gene expression from RNA profiles of intact tissues (30), to analyze various infiltrating immune cells in LGBLEL and CH using the FPKM values in expression profile microarray data through R Studio. As shown in Figure 4, a total of 18 and 17 major leukocyte subsets were revealed in LGBLEL (Figure 4A) in CH (Figure 4B), respectively. Among them, there were no monocytes in LGBLEL, while naïve B cells and activated CD4 + memory T cells were absent in CH. In addition, we focused on B lymphocyte-associated immune cells and found that memory B cell numbers were significantly higher in LGBLEL than in CH (Figure 4C) and the immunofluorescence results of CD27 showed that there were indeed memory B cells in LGBLEL (Figure 4D), which were significantly higher than CH (Figure 4E). LGBLEL had activated CD4 + T cells and high levels of memory B cells, indicating that an immune process is highly likely to occur in LGBLEL due to the characteristics of lymphocyte infiltration, mainly B lymphocytes. EcoTyper (31) was used to analyze and describe the state of LGBLEL epithelial cells and preliminarily explore the interaction between B cells and epithelial cells using expression profile microarray data. In total, 11 of our 16 LGBLEL microarray samples were enriched in 5 states of epithelial cell (Figure 4F); no enrichment was observed in state 3 (Pro-angiogenic), suggesting that epithelial cells in LGBLEL are immune to EMT-related tumor transformation, except for reduced proliferation. The cell–cell interaction network of different types and states is shown in Figure 4G (31), on the basis of which we screened genes interacting with ligands and epithelial cell receptors in B cells (Figure 4H). Among them, VIP (33), LTB (34), and IL-16 (35) are related to epithelial cell apoptosis, while CCL21 (36), CCL19 and WNT1 are related to tumor fibrosis. This result might provide preliminary data on the pattern of association between B cells and epithelial cells in LGBLEL.

### 3.5. Expression of proliferation and apoptosis related proteins in LGBELE tissue

**FIGURE 5:**
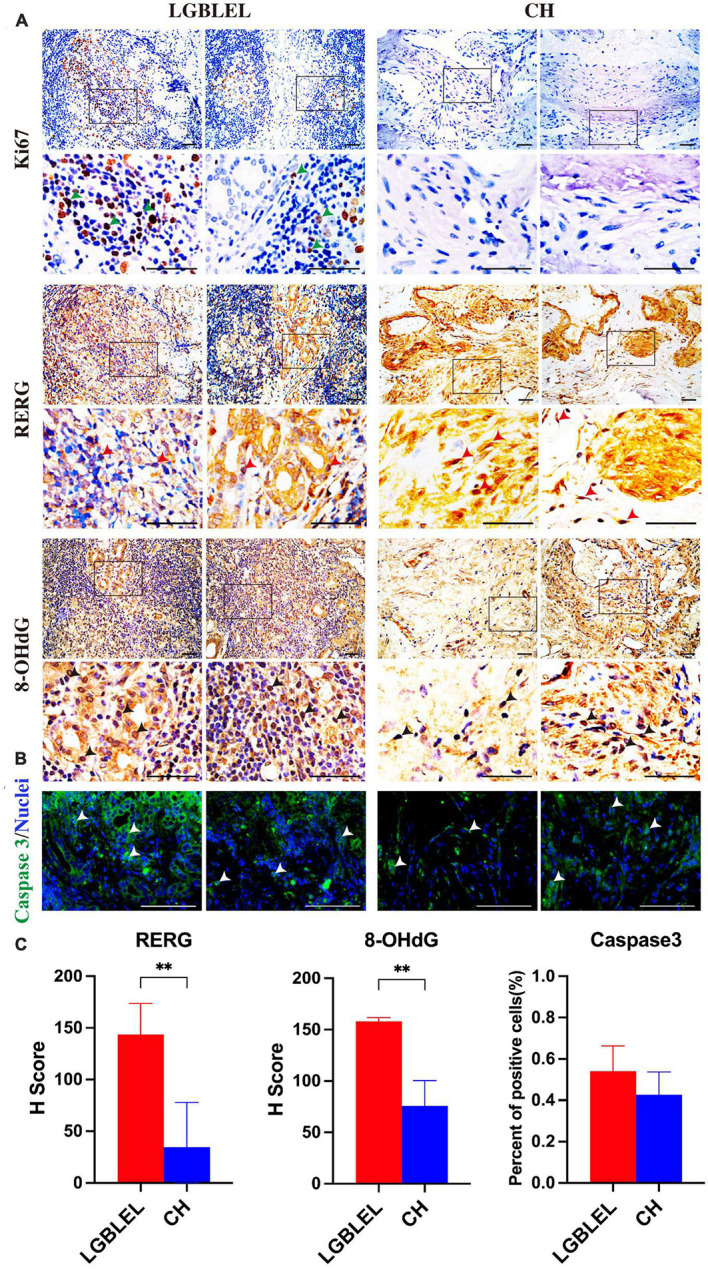
Analysis of cell proliferation and apoptosis characteristics of LGBLEL: **(A)** Immunohistochemistry results for Ki67 (green arrows), RERG (red arrows), and 8-OHdG (black arrows) in LGBLEL and control CH; **(B)** Immunofluorescence results of caspase 3 in LGBLEL and CH; **(C)** The quantitative determination of IHC and IF staining for RERG, 8-OHdG and Caspase 3. Scale bar: 20 μm. *P*-values were calculated using a Mann–Whitney test. **P < 0.01.

Because we observed that, in LGBLEL, increased expression of ERβ and estrogen receptor cell proliferation inhibitor genes, such as RERG RNA reduced and RERG, can inhibit cell proliferation and tumor formation (37), we next evaluated the effect of RERG on the regulation of lymphocyte infiltration and lacrimal gland atrophy in LGBLEL. As shown in Figure 5A, Ki67 was detected in the lymphocyte infiltration area of LGBLEL but not in the lacrimal glands. RERG is highly expressed in almost all LGBLEL lacrimal glands but relatively lower in the lymphocyte infiltration area (Figure 5B) and the H-Score of RERG in LGBLEL was significantly higher than that in CH (Figure 5D). Meanwhile, we measured the expression of 8-OHdG to explore the influence of ERβ and RERG on DNA damage in LGBLEL. As shown in Figure 5C, 8-OHdG positive cells (green arrow) were observed in atrophied lacrimal glands in LGBLEL, and only a few positive cells were found in lymphocyte infiltration areas; in the controls, 8-OHdG was also detected in some lacrimal cells in ERβ-negative LGB and in some fibrocytes in the intervascular fibrous connective tissues of CH (Figure 5C and Supplementary Figure 2). The H-Score of 8-OHdG in LGBLEL was significantly higher than that in CH (Figure 5D). Moreover, the results of immunofluorescence showed that caspase 3 was highly expressed in the lacrimal gland area of LGBLEL, but less in the lymphatic infiltration area. The CH group also had less caspase 3 expression (Figure 5C), but in general, there was no statistically significant difference between the two groups (Figure 5D).

Our results showed that ERβ and RERG are highly expressed in lacrimal glands. Previous studies have demonstrated that ERβ is related to lacrimal cell apoptosis and that RERG can inhibit cell proliferation. Additionally, DNA damage and apoptosis occurred in the lacrimal glands in LGBLEL, consistent with the results of most studies on lacrimal gland atrophy in LGBLEL. Ki67 was only expressed in lymphocyte infiltrating regions, consistent with the results of lymphocyte proliferation demonstrated in Figures 1, 2. These results are highly consistent with the characteristics of lacrimal gland parenchymal atrophy and lymphocyte infiltration in LGBLEL.

## 4. Discussion

Lacrimal gland benign lymphoepithelial lesion is an IgG4-related autoimmune disorder with unknown pathogenesis that primarily occurs in middle-aged women. Estrogen levels decrease with age, and it is hypothesized that the incidence of LGBLEL is related to an estrogen imbalance. The changes in the expression levels of estrogen and its receptors are often involved in the occurrence and invasiveness of tumors (38). This study expands our knowledge of estrogen receptors and, for the first time, sheds light on the relationship between LGBLEL pathological features and estrogen receptor levels, especially ESR2.

In this study, histological, serological, and transcriptomic analyses of clinical LGBLEL samples revealed that the expression pattern of ERβ was tissue-heterogeneous, with higher expression levels in the lacrimal gland region and lower expression levels in the area of lymphocyte infiltration. Accordingly, LGBLEL showed lacrimal gland atrophy accompanied by lymphocyte proliferation, consistent with the reported clinical manifestations. We also found that RERG, downstream of ERβ, exhibited a similar expression pattern and tissue heterogeneity and, thus, it potentially contributes to lacrimal gland cell apoptosis and lymphocyte proliferation.

The results of our study are not entirely consistent with the conventional view of the role of estrogen and its receptors in cancer. To date, in the study of diseases related to organs of the female reproductive system, many studies on estrogen and its receptors in breast cancer have revealed that sustained high levels of estrogen (endogenous or exogenous) can increase the incidence of breast cancer (39, 40). The main mechanism is that estrogen can promote epithelial cell proliferation (41), and growth and inhibit apoptosis by stimulating the expression of various growth factor genes mediated by abnormal estrogen signaling (42, 43). Additionally, estrogen metabolites can covalently bind to DNA and cause purine mutations that induce breast cancer (44). Similarly, other studies have shown that the lower the expression of ER is, the higher the degree of malignancy will be, as demonstrated by ER-negative breast cancer (45–47). The use of estrogen in postmenopausal women increases the risk of ovarian cancer (48), and the use of estrogen-lowering drugs such as aspirin can reduce this risk (49). The main mechanism is that estrogen induces endothelial cell proliferation and angiogenesis through ER-related pathways, thereby promoting the occurrence and progression of ovarian cancer (50). However, our results showed that high levels of ERβ and the downstream RERG induced epithelial cell apoptosis and lacrimal gland atrophy, while low levels of ERβ and RERG induced lymphocyte proliferation (Figures 1B, 2, 5). Meanwhile, different subtypes of ER have distinct effects on survival: ERβ is associated with tumor suppression and better survival outcomes (51, 52), whereas high expression of ERα is associated with poor survival outcomes (53, 54). This observation is also reflected in our results, as ERα is low or not expressed in LGBLEL, and the dominant location of ERβ (Figure 1B) appears to be one of the reasons that LGBLEL is a benign epithelial tumor.

The results of our study are similar to those found in studies on non-female genital diseases. Estrogen can inhibit the migration of gastric cancer cells by inhibiting MSCS (55) and NF-kB pathways to reduce the activity of gastric cancer cells and induce their apoptosis (56, 57). Both ERα and ERβ are expressed in gastric cancer and normal gastric tissues, but studies have shown that the physiological effect of estrogen in gastric cancer is mainly realized through ERβ-mediated inhibition of tumor cell proliferation and invasion (58). Positive ERα status seems to be related to tumor metastasis and invasion (59); however, there is also evidence that the overexpression of ERα can inhibit the proliferation of tumor cells *in vitro* (60). Similarly, reduced estrogen levels can significantly exacerbate arthritis (61), and ovariectomy in animal models studies can trigger Sjogren’s syndrome (62).

In our study of LGBLEL, we found that ERβ plays a dominant role in the orbit and is tissue-specific in the background of unchanged estrogen levels, with low expression in the lymphatic infiltration area and high expression in the lacrimal gland.

The proliferation of lymphocytes in LGBLEL is associated with ERβ, and there is evidence that the proliferation of IgG4-secreting plasma cells can lead to local tissue fibrosis and sclerosis (63). Long-term fibrosis will lead to the surrounding lacrimal gland gradually occupying. High ERβ expression in the lacrimal gland area will inhibit the proliferation of lacrimal gland cells, causing lacrimal gland atrophy.

Our study, to some extent, indicates the evolution of LGBLEL. Although LGBLEL is normally a benign inflammatory hyperplastic disease, a small proportion (12.0–14.3%) of LGBLEL develops into a malignant tumor, according to the literature and clinical evidence (2). The terminal stage of the evolved malignant tumor is usually MALT (64), which is related to B lymphocyte proliferation (65). We found that the upregulated genes in LGBLEL were highly enriched in the cell proliferation-associated GO clusters (Figures 2A–C), of which lymphocytes, especially B cells, were highly proliferating and infiltrating (Figures 4A, 5) in the affected lacrimal tissue. However, the lacrimal gland cells underwent apoptosis processes (Figures 4D, F, 5) with no signs of EMT (Figure 4D), indicating a low incidence of malignant epithelial transformation (66, 67). Moreover, in the preliminary exploration of the interaction pattern between B cell and epithelial cell populations in LGBLEL, it was found that certain B cell ligands can interact with receptors on epithelial cells, potentially leading to further lymphocyte proliferation and gland atrophy (Figure 4F). These histological and transcriptome features matched the clinical evidence of inflammatory swelling. Over time, the infiltrating lymphocytes enhance the space occupation of the lacrimal gland and promote lacrimal atrophy. Therefore, the heterogeneity expression of ERβ/RERG could trigger LGBLEL tumor development.

Notably, among the proliferating lymphocytes, the cell proportions of memory B cells and plasma B cells were much higher in the LGBLEL group than in the control group in the immune cell subpopulation analysis (Figures 4A, B). Long-term humoral immunity depends on high-affinity class-switched memory B cells and long-lived antibody-secreting plasma B cells, both of which can produce IgG (68). Thus, the ERβ/RERG-associated B cell proliferation in LGBLEL could potentially explain the origin of this IgG4-related autoimmune orbital disease.

In some epithelial tumors, RERG inhibits tumor cell proliferation, growth, migration, invasion, and angiogenesis by inhibiting the ERK/NF-κB signaling pathway (69). These effects are dependent on estrogen receptors, and ERβ has been found to be enriched in the RERG promoter region (70). In our results, consistency between ERβ and RERG expression levels in tissues was clearly observed and was found to be highly correlated with lacrimal gland cell apoptosis (Figures 1B, 5).

**FIGURE 6:**
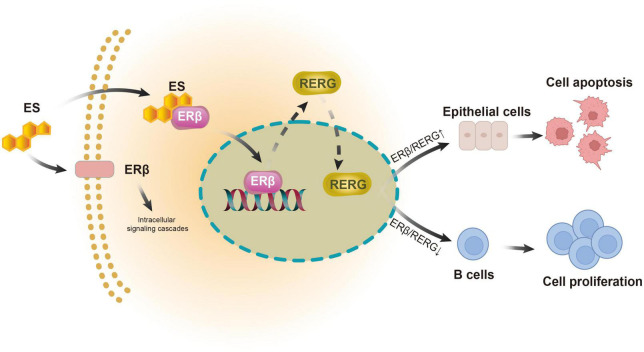
Models of action of ERβ and RERG in epithelial cells and B cells in LGBLEL.

In summary, we demonstrated the tissue-heterogeneous expression pattern of ERβ and its downstream receptor RERG in LGBLEL for the first time. Both estrogen receptors exhibit higher expression levels in the lacrimal gland and lower expression levels in the lymphatic infiltration region, potentially correlating with the clinical outcomes of inflammatory swelling and lacrimal gland atrophy in LGBLEL (Figure 6). The histological results were confirmed by the transcriptome sequencing data; the upregulated genes were enriched in lymphocyte proliferation-related functions, while the epithelial cells were not proliferative. Further data mining revealed that memory B cells and plasma B cells were the most proliferative among all the immune cells, and no EMT was observed in the epithelial tissue of LGBLEL. The gene expression correlation analysis revealed that lacrimal gland apoptosis could be attributed to the B cell infiltration. Although more investigations are still needed to verify the functions and mechanisms of ERβ and RERG in LGBLEL development, we believe that the findings presented here shed light on the potential mechanisms of LGBLEL evolution and progression, providing guiding significance for the clinical diagnosis and treatment of LGBLEL and other IgG4-related diseases.

## Data availability statement

Publicly available datasets were analyzed in this study. This data can be found here: https://www.ncbi.nlm.nih.gov/geo/query/acc.cgi?acc=GSE76497: GSE76497.

## Ethics statement

The study was conducted in accordance with the Declaration of Helsinki, and approved by the Local Ethics Committee of Beijing Tongren Hospital, Capital Medical University (CHINA, proto-col code TRECKY203-KS-05 and date of approval 28 February 2013). A signed consent was obtained from each patient prior to blood and tissues collection. Written informed consent has been obtained from the patients to publish this paper. Written informed consent to participate in this study was provided by the participants’ legal guardian/next of kin.

## Author contributions

XZ and PZ: conceptualization and writing–original draft. MM, HW, and RL: data curation. XZ, PZ, MM, HW, RL, ZL, and ZC: formal analysis and methodology. PZ and XM: funding acquisition. XZ: visualization. XZ, ML, FX, and XM: writing–review and editing. All authors have read and agreed to the published version of the manuscript.
