# Supplementary figures and images for: Missing link between tissue specific expressing pattern of ERβ and the clinical manifestations in LGBLEL
Source: Front Med (Lausanne). 2023 Jun 29;10:1168977. doi: 10.3389/fmed.2023.1168977 (PMC10346852; doi:10.3389/fmed.2023.1168977)

**A**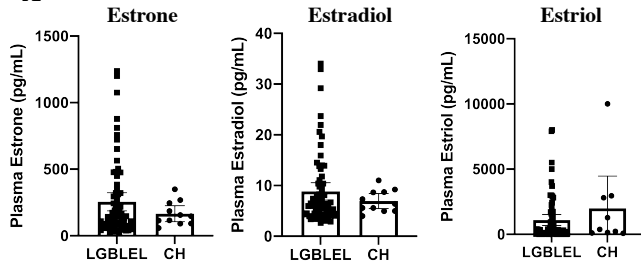**B**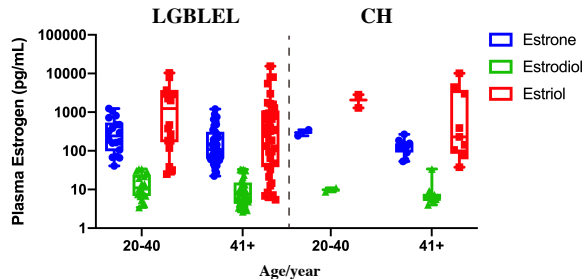**C**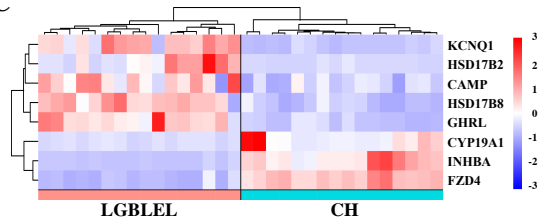**D**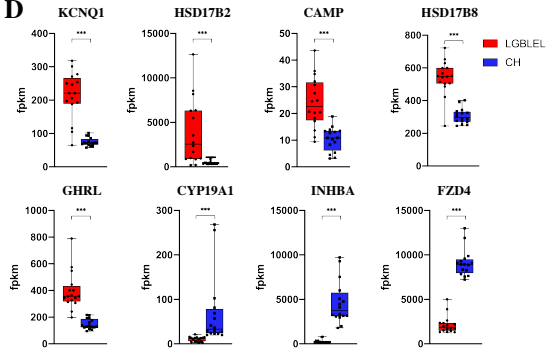

Supplement: Supplementary file 1 [file Data_Sheet_1.ZIP › Supplement figure S1 plasma estrogen levels.pdf]

**ER $\alpha$**

**ER $\beta$**

**RERG**

**Ki67**

**LGB**

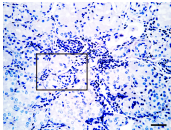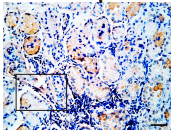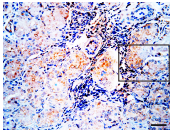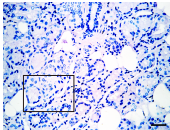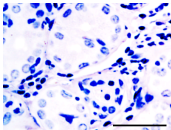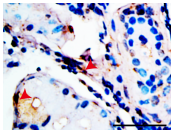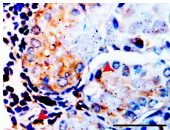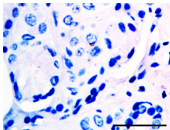

Supplement: Supplementary file 1 [file Data_Sheet_1.ZIP › Supplementary figure S2 LGB IHC.pdf]

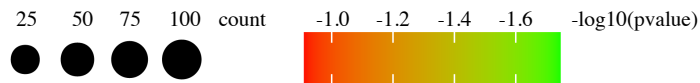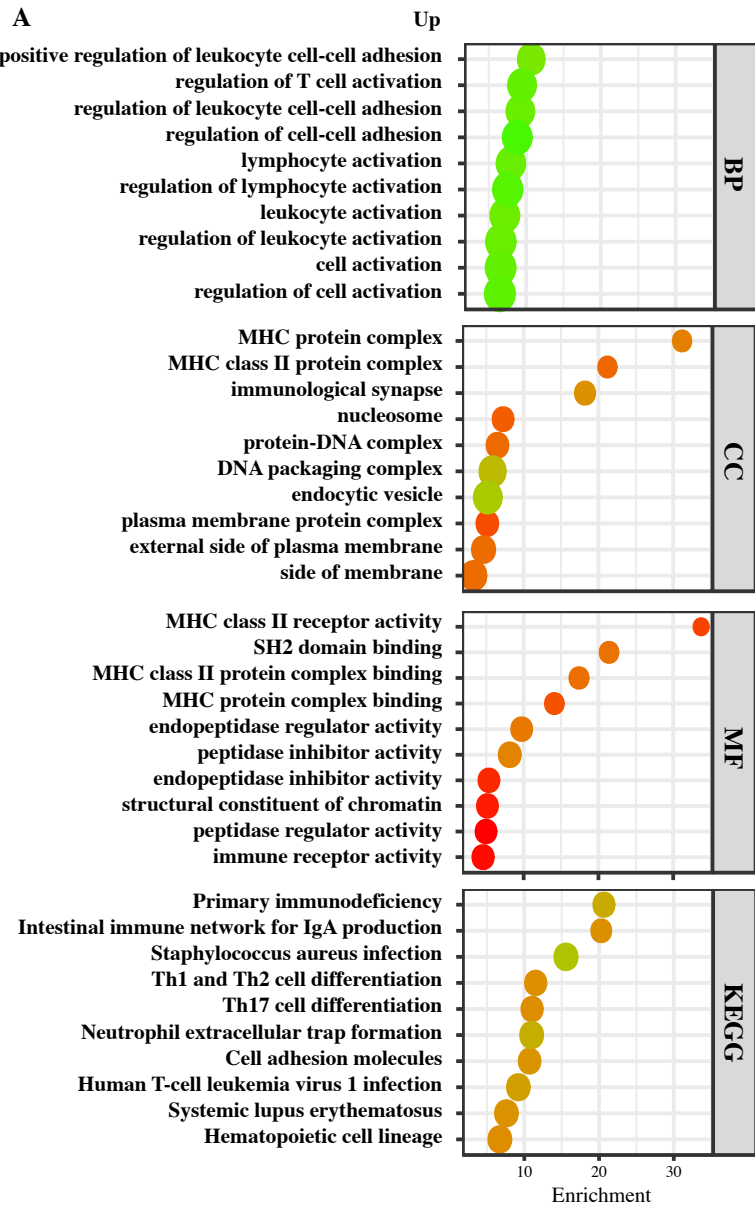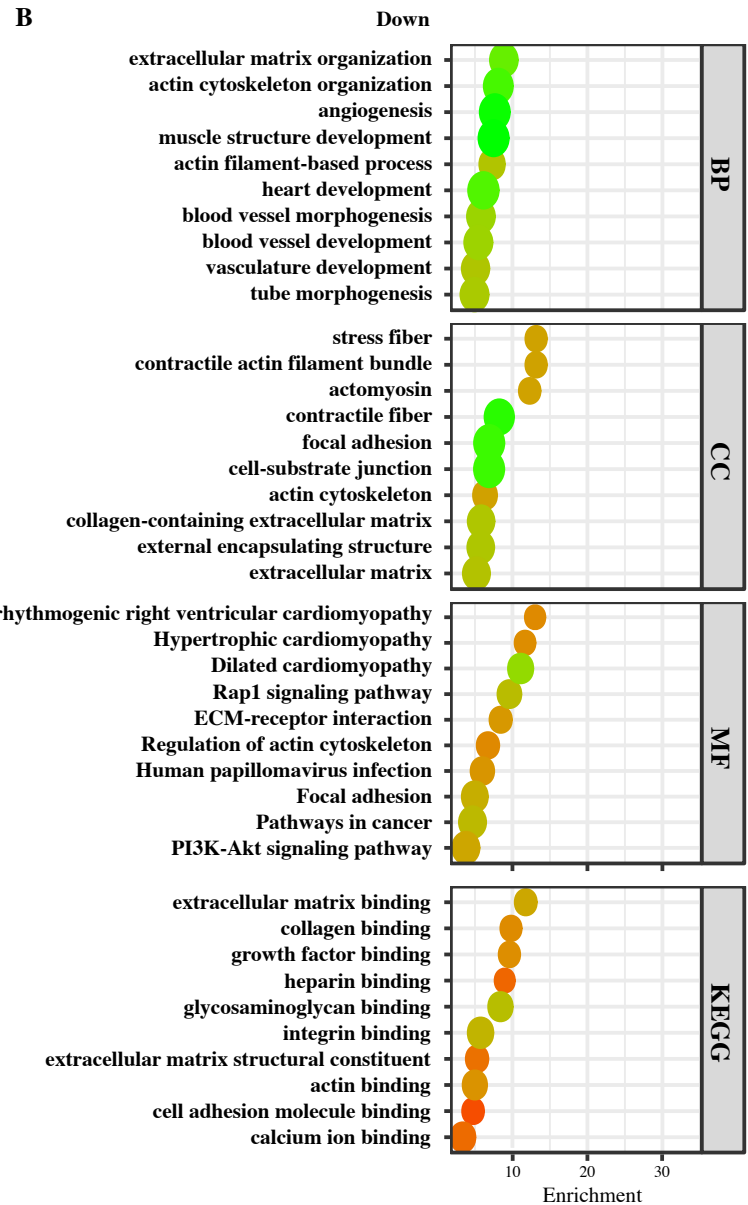

Supplement: Supplementary file 1 [file Data_Sheet_1.ZIP › Supplementary figure S3 GO kegg.pdf]

Up

KEGG

GO:BP

GO:MF

GO:CC

Down

KEGG

GO:BP

GO:MF

GO:CC

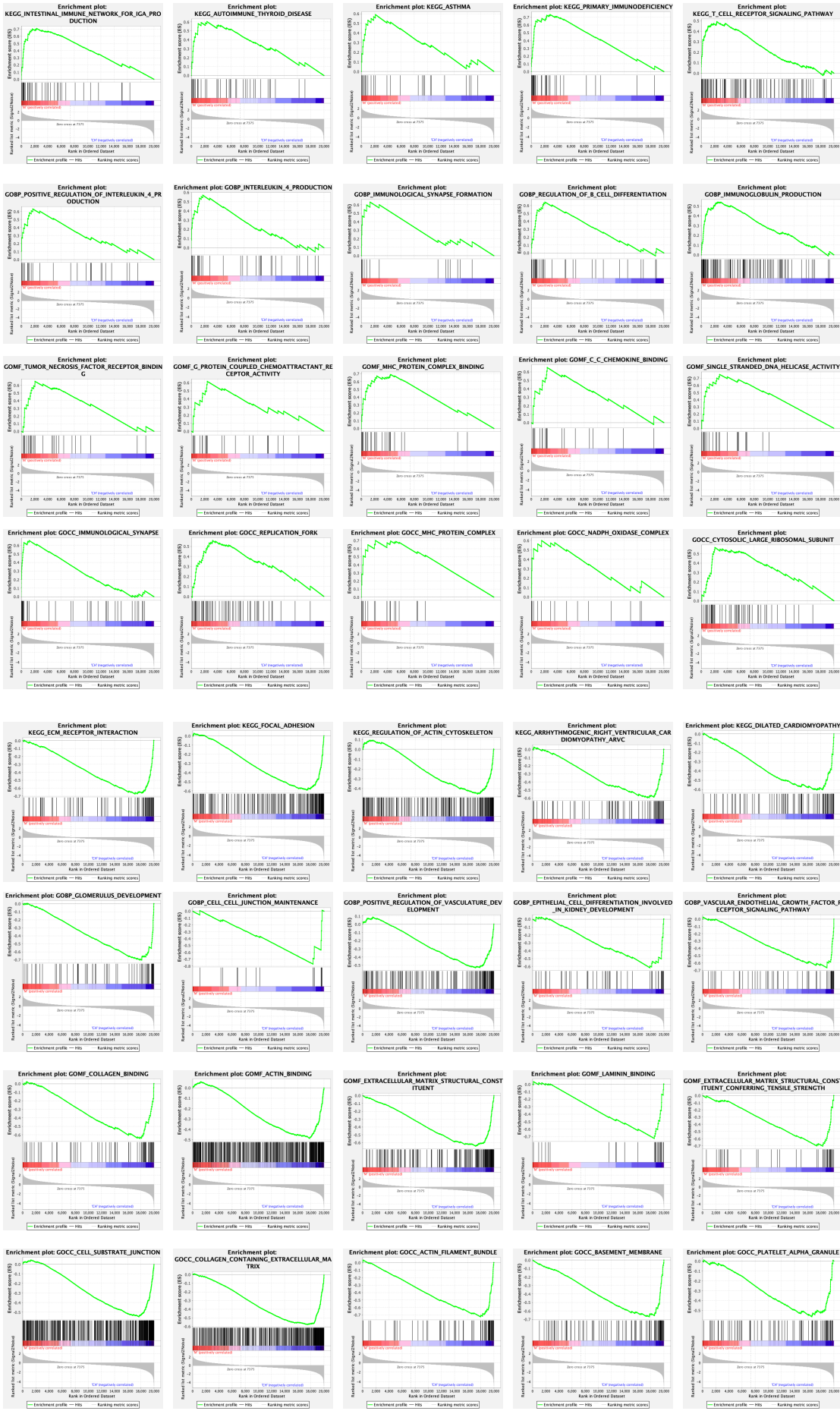

Supplement: Supplementary file 1 [file Data_Sheet_1.ZIP › Supplementary figure S4.pdf]
